# Amputation of a Healthy Upper Jaw, as a Preliminary Step to the Removal of a Fibro-Plastic Tumor of the Pharynx

**Published:** 1853-04

**Authors:** 


### ARTICLE XVIII.

### *Amputation of a Healthy Upper Jaw, as a preliminary step to the Removal of a Fibro-plastic Tumor of the Pharynx.*

M. Maisonneuve presented to the Academy of Medicine of Paris, at the end of the meeting of the 20th of April, 1852, a patient upon whom he had performed the amputation of a healthy superior maxilla, in order to render practicable the extirpation of a fibro-plastic tumor situated in the pharynx, with polypoid extensions into the nasal fossæ, the frontal sinuses, the temporal fossa, the zygomatic space, and the cheek. The operation was performed in such a manner that the incisions carried along the mesial line, and the commissure of the lips, did not divide any motor nerve. The movements of the face are thus perfectly preserved, and no deformity is visible. When the wound was quite healed up, M. Maisonneuve had an apparatus constructed to which the teeth of the patient were adapted; this apparatus comes, therefore, to answer the purpose of an obturator and a set of teeth. Mastication and articulation are performed as well as in the normal state, thanks to this mechanical contrivance, and the loss which this patient has undergone is thoroughly concealed.

